# Efficacy and Safety of Anti-Interleukin-5 Therapy in Patients with Asthma: A Systematic Review and Meta-Analysis

**DOI:** 10.1371/journal.pone.0166833

**Published:** 2016-11-22

**Authors:** Fa-Ping Wang, Ting Liu, Zhu Lan, Su-Yun Li, Hui Mao

**Affiliations:** 1 Department of Respiratory Medicine, West China Hospital, Sichuan University, Chengdu, 610041, China; 2 Department of Gynecology and Obstetrics, West China Second University Hospital, Sichuan University, Chengdu, 610041, China; 3 Department of Respiratory Medicine, First Affiliated Hospital of Henan College of Traditional Chinese Medicine, Zhengzhou, 450000, China; National and Kapodistrian University of Athens, GREECE

## Abstract

**Background:**

Recent trials have assessed the efficacy and safety of novel monoclonal antibodies such as reslizumab and benralizumab. However, the overall efficacy and safety anti—interleukin (IL) 5 treatment in asthma have not been thoroughly assessed.

**Methods:**

Randomized controlled trials (RCTs) of anti-IL-5 treatment on patients with asthma published up to October 2016 in PubMed, Embase, and Cochrane Central Register of Controlled Trials (CENTRAL) that reported pulmonary function, quality of life scores, asthmatic exacerbation rate, blood and sputum eosinophil counts, short-acting β-agonist (SABA) rescue use, and adverse events were included. The pooled mean difference, and relative risks (RR), and 95% confidence intervals (CIs) were calculated using random-effects models.

**Results:**

Twenty studies involving 7100 patients were identified. Pooled analysis revealed significant improvements in FEV_1_ (first second forced expiratory volume) (MD = 0.09, 95% CI: 0.06–0.12, *I*^*2*^ = 10%), FEV_1_% (MD = 3.75, 95% CI: 1.66–5.83, *I*^*2*^ = 19%), Asthma Quality of Life Questionnaire (AQLQ) score (MD = 0.22, 95% CI: 0.15–0.30, *I*^*2*^ = 0%), decreased blood, sputum eosinophils and asthmatic exacerbation (RR = 0.66, 95% CI: 0.59–0.73, *I*^*2*^ = 51%); peak expiratory flow (PEF) (MD = 5.45, 95% CI: -2.83–13.72, *I*^*2*^ = 0%), histamine PC_20_ (MD = -0.62, 95% CI: -1.92–0.68, *I*^*2*^ = 0%) or SABA rescue use (MD = -0.11, 95% CI: -0.3–0.07, *I*^*2*^ = 30%) were unaffected; adverse events were not increased (RR = 0.93, 95% CI: 0.89–0.98, *I*^*2*^ = 46%). No publication bias was observed (Egger's *P* = 0.78).

**Conclusions:**

Anti-interleukin 5 monoclonal therapies for asthma could be safe for slightly improving FEV_1_ (or FEV_1_% of predicted value), quality of life, and reducing exacerbations risk and blood and sputum eosinophils, but have no significant effect on PEF, histamine PC20, and SABA rescue use. Further trials required to establish to clarify the optimal antibody for different patients.

## Introduction

Asthma is a common chronic inflammatory disease that affects more than 300 million people worldwide, and imposes a high disease burden and economic impact globally [1–3]. Despite taking high-dosage inhaled corticosteroids according to the Global Initiative for Asthma (GINA) guidelines, at least 40% of patients continue to suffer from inadequately controlled symptoms, either because they are truly resistant or because they do not take them [4, 5]. Patients who remain uncontrolled can use other drugs such as leukotriene-receptor antagonists, slow-release theophylline, and long-acting anticholinergics [6]. Since the anti-immunoglobulin (Ig)E humanized monoclonal antibody omalizumab became the first biological treatment approved for treating allergic asthma, many small molecules and monoclonal antibodies targeting biomolecular specificities have been investigated for treating symptomatic asthma [7]. Eosinophilic inflammatory infiltration is a central event in asthma pathogenesis. IL-5 is the chief cytokine responsible for eosinophil production, survival, maturation and recruitment and activation at allergic inflammation sites [8]. Preclinical studies have demonstrated a key role for IL-5 in murine models of allergen-induced airway eosinophilia and hyperresponsiveness [9]. Given the relationship of IL-5 to eosinophilia and asthma severity, human(ized) monoclonal antibodies targeting IL-5 have shown great promise in severe refractory asthma with persistent eosinophilia [10, 11]. The anti-IL-5 agents benralizumab, reslizumab, and mepolizumab have been investigated for treating asthma [12, 13]. However, their effects on lung function (especially FEV_1_) have been less consistent. Here, we conducted a meta-analysis of randomized, controlled trials (RCTs) to assess whether anti-IL-5 monoclonal antibodies therapy is safe and effective in patients (more than 12 years) with asthma.

## Methods

### Literature searches and study selection

PubMed, Embase, and the Cochrane Central Register of Controlled Trials (CENTRAL) were searched for articles published from 1946 to October 2016, using the search terms: ‘‘anti–interleukin-5” or ‘‘mepolizumab” or “benralizumab” or “reslizumab” or “monoclonal antibody” or “anti-IL-5”, combined with ‘‘asthma”. Language restrictions were not applied. Reviews and the reference lists of relevant articles were also screened for additional articles of interest. Two independent authors (FPW and TL) screened all references according to the selection criteria. To ensure a complete review of the available studies, the abstracts of relevant scientific meetings were also examined, but trials published solely in abstract form were excluded. Any disagreements were resolved by consensus with a third author when necessary. The details of the search strategy are displayed in S1 Table.

### Inclusion and exclusion criteria

Eligible clinical trials were defined as: (1) adults/adolescents (≥12 years) with diagnosis of asthma; (2) investigations of patients who received anti-interleukin-5 monoclonal antibody therapy at any dose, placebo-controlled or standard therapy; (3) randomized (parallel group) placebo-controlled trials, and (4) RCTs reporting the following outcomes: blood and sputum eosinophil count, asthma exacerbation, lung function, asthma control and quality of life scores, rescue use of SABA and adverse events. We excluded non-randomized, observational, cohort, case-control and non-blinded clinical trials. FPW and TL independently screened all references according to the selection criteria. Differences in opinion about inclusion were resolved by mutual agreement and arbitration of a third author (HM).

### Data extraction and quality assessment

FPW and TL independently extracted related data in blinded fashion from eligible studies based on the predefined criteria, which included the characteristics of the trials, interventions, and outcomes. The predefined primary outcomes were lung function [first second forced expiratory volume (FEV_1_), FEV_1_% of predicted value, peak expiratory flow (PEF), histamine PC_20_], the Asthma Quality of Life Questionnaire (AQLQ) scores, and asthma exacerbation. Asthma exacerbation was defined as a worsening of asthma requiring increased corticosteroids or albuterol dose to control symptoms and/or the need for asthma-related emergency treatment/hospitalization. Secondary outcomes were adverse events and efficacy outcomes [blood eosinophil count, sputum eosinophils (%), short-acting β-agonist (SABA) rescue use]. The risk of bias was assessed using Cochrane-recommended tools, which included: (1) adequate sequence generation; (2) allocation concealment; (3) blinding; (4) incomplete outcome data addressed; (5) free of selective reporting; and (6) free of other bias [14].

### Statistical analyses

All analyses were performed with Review Manager (Version 5.3, The Cochrane Collaboration) and Stata (Version 12.0, Stata Corporation, USA), *P* <0.05 was considered statistically significant. If a study presented more than two interventions, we combined two or three intervention groups into a single intervention group in accordance with the Cochrane handbook.^9^ Random-effects model was applied in all data analyses regardless of statistical heterogeneity. Risk ratio (RR) and 95% CIs were used to analyze dichotomous data, and mean difference and 95% CI were used for continuous data. Heterogeneity assumptions were assessed using the *I*^*2*^ statistic (*I*^*2*^>50% indicates significant heterogeneity), and tested with the χ^2^ statistic (*P*<0.05). However, the number of studies affects both the power of the heterogeneity test and the heterogeneity measures *I*^*2*^, but not $H_{M}^{2}$ [15]. In order to the increase the power of detecting heterogeneity, the 95% CI of *I*^*2*^ and $H_{M}^{2}$ were calculated [15–17]. If substantial heterogeneity was identified, subgroup and sensitivity analyses were performed. Moreover, we separately performed subanalyses in different drugs for each outcome. Publication bias was determined using the Begg’s funnel plot and assessed by Egger’s test if the number of the studies was larger than ten.

## Results

### Study characteristics

**Fig 1:**
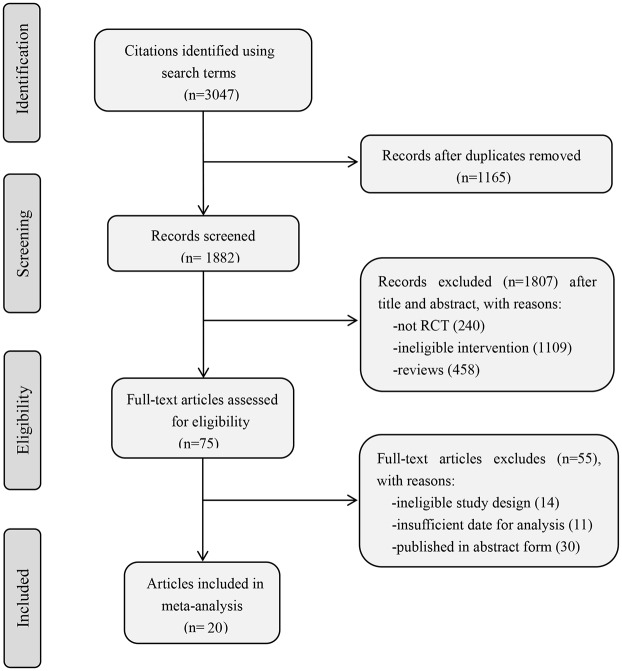
Flow chart of study identification, inclusion, and exclusion.

We identified 3047 manuscripts: 2019 from PubMed, 893 from Embase, 135 from CENTRAL. Based on title/abstract and full-text screening, 20 RCTs were included in the meta-analysis. Fig 1 summarized the study selection process [18–37].

Tables 1–3 lists the RCT characteristics, and Table 4 describes the baseline characteristics of the patients enrolled. Sample sizes ranged from 19 to 1306 subjects. Nine, five, and six trials used mepolizumab [18–26], reslizumab [27–31], and benralizumab [32–37], respectively. Treatment duration ranged from 1 day to 56 weeks and follow-up ranged from 12 to 56 weeks. Seven studies administered drugs used subcutaneous injection [25, 26, 32, 33, 35–37], while the remaining studies used intravenous infusion [18–24, 27–31, 34]. Nine studies involved patients with severe/refractory asthma [22–28, 36, 37]; four studies included patients with mild, mild to moderate, or moderate asthma [18–21]; the remaining studies did not specify asthma severity [29–35]. Corren et al. [30] and Castro et al. [33] studied patients with non-eosinophilic asthma.

**Table 1:** Characteristic of randomized controlled trials included.

Reference	Study Design	No. of Subjects	Population	Age	drug	dosing	Outcomes	Follow-up	Exacerbation definition
Leckie 2000^[18]^	multi-center, double-blind	24	mild atopic asthma	18–45	Mepolizumab	Single IV dose of 2.5 or 10 mg/kg or placebo for one day	Blood and sputum eosinophils; histamine PC_20;_	16 weeks	NM
Flood-Page PT 2003^[19]^	Two-center, double-blind parallel	24	mild atopic asthma	18–55	Mepolizumab	Three IV doses of 750 mg or placebo for 8 weeks	Blood eosinophils;FEV_1_; PEFR; histamine PC_20_	20 weeks	NM
Büttner 2003^[20]^	multi-center, double-blind	19	mild or moderate asthma with ICS	20–59	Mepolizumab	Three IV doses of 250, 750 mg or placebo for 12 weeks	Blood eosinophils	6 months	NM
Flood-Page P 2007^[21]^	multi-center, double-blind	362	moderate persistent asthma with ICS	18–55	Mepolizumab	Three IV doses of 250, 750 mg or placebo for 12 weeks	Blood and sputum eosinophils;FEV_1_; PEF; symptom scores; asthma exacerbations	20 weeks	An acute worsening of asthma requiring additional treatment in excess of an increase in short-acting β_2_-agonist.
Haldar 2009^[22]^	Single-center doubleblind,paralle	61	refractory eosinophilic asthma	18–72	Mepolizumab	Twelve IV doses of 750 mg or placebo for 50 weeks	Blood and sputum eosinophils;FEV_1_; AQLQ; JACQ; FENO; histamine PC_20_;asthma exacerbations	50 weeks	Periods of deterioration in asthma control in subjects who had been treated with high-dose oral prednisolone for at least 5 days
Nair 2009^[23]^	Single-center, double-blind, paralle	20	severe asthma on OCS with persistent sputum eosinophilia	NM	Mepolizumab	Five IV doses of 750 mg or placebo for 16 weeks	Blood and sputum eosinophils;FEV_1_;JACQ; asthma exacerbations; reduction in the dose of prednisone	26 weeks	A patient initiated increase in the daily dose of albuterol of four or more puffs to control symptoms of chest tightness or as any one of the following: nocturnal or waking respiratory symptoms on two consecutive days, a decrease of more than 15% in the FEV_1_ from the level at randomization after the use of albuterol, or a 2-point worsening in the Likert score for cough by the investigators at their discretion on the basis of general clinical worsening.
Pavord 2012^[24]^	multi-center, double-blind	621	severe eosinophilic asthma	12–74	Mepolizumab	Thirteen IV doses of 75, 250, or 750 mg or placebo for 52 weeks	Blood and sputum eosinophils;FEV_1_; AQLQ; ACQ-6;asthma exacerbations	52 weeks	Worsening of asthma requiring use of oral corticosteroids for 3 or more days, admission, or a visit to the emergency department, 50% increase in rescue medication on at least 2 of 3 successive days, increased frequency of nocturnal awakening due to asthma for at least 2 of 3 successive nights, or overall asthma symptom score of five (scale one to five) for at least 2 of 3 successive days
Bel 2014^[25]^	multi-center doubleblind, parallel	135	severe eosinophilic asthma	16–74	Mepolizumab	Six SC doses of 100 mg or placebo for 20 weeks	Asthma exacerbations; ACQ-5;SGRQ; reduction in the oral glucocorticoid dose	32 weeks	A worsening of asthma leading to the doubling (or more) of the existing maintenance dose of oral glucocorticoids for 3 or more days or hospital admission or an emergency department visit for asthma treatment
Ortega 2014^[26]^	multi-center double-blind,	576	severe eosinophilic asthma	12–84	Mepolizumab	Nine doses of 75 mg IV or 100 mg SC or placebo for 32 weeks	Asthma exacerbations; ACQ-5;SGRQ;FEV1;blood eosinophil	40 weeks	Worsening of asthma such that the treating physician elected to administer systemic glucocorticoids for at least 3 days or the patient visited an emergency department or was hospitalized
Kips JC 2003^[27]^	multi-center, double-blind	32	severe persistent asthma	NM	Reslizumab	Single IV doses of 0.03mg/kg,0.1mg/kg, 0.3mg/kg, or 1mg/kg or placebo for one day	Blood and sputum eosinophils; FEV_1_;	90 days	NM

**Table 2:** Characteristic of randomized controlled trials included.

Reference	Study Design	No. of Subjects	Population	Age	drug	dosing	Outcomes	Follow-up	Exacerbation definition
Castro 2011^[28]^	multi-center, double-blind	106	severe eosinophilic asthma	18–75	Reslizumab	Three IV doses of 3mg/kg or placebo for 12 weeks	Blood and sputum eosinophils;ACQ-7; FEV_1_; asthma exacerbations	15 weeks	A 20% or more decrease from baseline in FEV_1_; or worsening of Asthma requiring emergency treatment, hospital admission, or three or more days of oral corticosteroid treatment.
Castro 2015^[29]^	two duplicate multi-center, double-blind parallel	Study 1:489 Study 2:464	uncontrolled asthma	12–75	Reslizumab	Thirteen IV doses of 3mg/kg or placebo for 52 weeks	Blood eosinophils; asthma exacerbations; FEV_1_; AQLQ; ACQ-7; rescue SABAs	52 weeks	Worsening of asthma that resulted in use of systemic corticosteroids in patients not already receiving treatment, or a two-times increase in the dose of either inhaled corticosteroids or systemic corticosteroids for 3 or more days, or the need for asthma-related emergency treatment
Corren 2016^[30]^	multi-center, double-blind	492	non-eosinophilic asthma	18–65	Reslizumab	Four IV doses of 3mg/kg or placebo for 16 weeks	Blood eosinophils;ACQ-7; FEV_1_;rescue SABAs	28 weeks	NM
Bjermer L 2016^[31]^	multi-center, double-blind parallel	315	uncontrolled asthma	12–75	Reslizumab	Four IV doses of 0.3mg/kg, 3mg/kg or placebo for 16 weeks	Blood eosinophils; FEV_1_; FVC;ACQ-6(5);FEF_25-75%_; ASUI; AQLQ	20 weeks	A reduction in FEV_1_ of ≥20%, hospitalization due to asthma, emergency treatment of asthma, or use of systemic corticosteroids for ≥3 days
Laviolette 2013^[32]^	multi-center, double-blind	cohort 1:13 cohort 2:14	eosinophilic asthma	18–65	Benralizumab	Single IV dose of 1mg/kg or placebo (cohort 1) for one day or three SC doses of 100 or 200 mg or placebo (cohort 2) for 56 days	Blood, sputum eosinophils; adverse events	84 days or 140 days	NM
Castro 2014^[33]^	multi-center, double-blind	group 1:324 group 2:282	group 1: eosinophilic asthma group 2: non-eosinophilic asthma	18–75	Benralizumab	Eight SC doses of 2, 20, or 100 mg for eosinophilic patients and 100 mg for non-eosinophilic or placebo for 1 year	Blood eosinophils; asthma exacerbations; FEV_1_; AQLQ;	1 year	An increase in asthma symptoms that did not resolve after rescue medication and needed treatment with systemic steroids for at least 3 days
Nowak 2015^[34]^	multi-center double-blind, parallel	110	acute asthma	18–60	Benralizumab	Single IV dose of 0.3mg/kg or 1mg/kg or placebo for one day	Blood eosinophils; asthma exacerbations; FEV_1_; ACQ-7; AQLQ	168 days	An increase of asthma symptoms that did not resolve within 2 hours after the use of rescue albuterol or corticosteroids and required an unscheduled medical visit; or during a scheduled study visit, the subject had acute asthma symptoms and a reduction of greater than or equal to 20% in predicted peak expiratory flow or FEV_1_, which in the opinion of the investigator required treatment.
Park HS 2016^[35]^	multi-center, double-blind	106	eosinophilic asthma	20–75	Benralizumab	Seven SC doses of 2, 20, or 100 mg or placebo for 40 weeks	Blood eosinophils; asthma exacerbations; FEV1; PEF; ACQ-6;FENO	52 weeks	An increase in asthma symptoms that required treatment with systemic steroids for at least 3 days.
Bleecker E R 2016^[36]^	multi-center, double-blind parallel	1306	severe uncontrolledasthma with eosinophilia	12–75	Benralizumab	Twelve SC doses of 30 mg or Seven SC doses of 30mg or placebo for 48 weeks	Asthma exacerbations; FEV1; ACQ-6; AQLQ	48 weeks	A worsening of asthma that led to one of the following: (1) use of systemic corticosteroids, or temporary increase in a stable oral corticosteroid background dosage, for at least 3 days or a single injectable dose of corticosteroids; (2) emergency department or visit to an urgent care centre (<24 h) because of asthma that needed systemic corticosteroids;or (3) inpatient hospital stay (≥24 h) because of asthma

**Table 3:** Characteristic of randomized controlled trials included.

Reference	Study Design	No. of Subjects	Population	Age	drug	dosing	Outcomes	Follow-up	Exacerbation definition
FitzGerald J M 2016^[37]^	multi-center, double-blind parallel	1205	severe,uncontrolled, eosinophilic asthma	12–75	Benralizumab	Fourteen SC doses of 30mg or Eight SC doses of 30mg or placebo for 56 weeks	Asthma exacerbations; FEV1; ACQ-6; AQLQ	56 weeks	An asthma exacerbation was defined as a worsening of asthma that led to one of the following: (1) use of systemic corticosteroids for 3 days or more or a temporary increase in a stable, background dosage of oral corticosteroids; (2) an emergency department or urgent care visit (<24 h) due to asthma that required systemic corticosteroids; or (3) an inpatient admission to hospital (≥24 h) due to asthma.

FEV1, forced expiratory volume in 1 second; PEF, peak expiratory flow; histamine PC20, provocative concentration of histamine required to cause a 20% fall in FEV1; JACQ, Juniper Asthma Control Questionnaire; AQLQ, the Asthma Quality of Life Questionnaire; ACQ, Asthma Control Questionnaire; FeNO, fraction of exhaled nitric oxide; SABAs: short-acting beta-agonists (SABAs); SC: subcutaneous injections; IV, intravenous; ICS, inhaled corticosteroid; OCS, oral corticosteroid; NM: not mentioned

**Table 4:** Baseline Characteristics of Patients in the 20 Studies Included.

Refs.	No. *	Sex	Age	Blood Eosinophils	FEV_1_% Predicted	Diagnosis of asthma	PEF
		(Male, %)*	(Mean SD, y)*	(Mean SD,10^3^/uL)*	(Mean SD, %) *	(mean SD, y)*	(Mean SD, L/min)*
Leckie 2000^[18]^	16	NM	29 (6.29)	0.25 (0.1)	86.15 (9.58)	NM	NM
Flood-Page PT 2003^[19]^	11	9 (82)	31 (5.5)	0.27 (0.18)	87.0 (6.3)	NM	433 (37.8)
Büttner 2003^[20]^	12	5 (42)	44.25 (10.85)	NM	65.68 (10.48)	11.75 (9.27)	NM
Flood-Page P 2007^[21]^	236	112 (47)	36 (29.4)	0.35 (0.25)	68.35 (9.2)	NM	366.6 (90.0)
Haldar 2009^[22]^	29	14 (48)	48 (7)	0.32 (0.38)^&^	78.1 (20.9)	NM	NM
Nair 2009^[23]^	9	4 (44)	56.4 (10.9)	0.68 (0.52)	66.6 (18.3)	NM	NM
Pavord 2012^[24]^	461	171 (37)	49.4 (11.2)	0.24 (1.03)^#^	60.0 (16.3)	19.5 (14.4)	NM
Bel 2014^[25]^	69	25 (36)	50 (9.7)	0.25 (1.245)^#^	59.6 (17.0)	17.4 (11.8)	NM
Ortega 2014^[26]^	385	163 (42)	50.5 (11.5)	0.285 (1.018)^#^	60.3 (17.9)	20.2 (13.4)	262 (110)
Kips JC 2003^[27]^	18	12 (67)	43 (5.9)	0.26 (0.04)	53.4 (7.6)	NM	NM
Castro 2011^[28]^	53	19 (36)	44.9 (13.94)	NM	66.0 (15.16)	23.3 (11.38)	NM
Castro 2015^[29]^	Study 1: 245 Study 2: 232	Study 1: 103 (42) Study 2: 88 (38)	Study 1: 48 (14.1) Study 2: 48 (14.4)	Study 1:0.696 (0.768) Study 2: 0.61 (0.412)	Study 1:63.6 (18.6) Study 2:70.4 (21.0)	Study 1:19.7 (15.2) Study 2:18.2 (14.4)	NM
Corren 2016^[30]^	398	137 (34)	44.9	0.281 (0.264)	66.8	26.2	NM
Bjermer L 2016^[31]^	210	85 (40)	43.7	0.65 (0.006)	69.6	20.2	NM
Laviolette 2013^[32]^	cohort 1: 8 cohort 2: 9	cohort 1: 6 (25) cohort 2: 5 (56)	cohort 1: 38.9 (14.7) cohort 2: 38.9 (13.8)	NM	cohort 1: 70.5 (15.6) cohort 2: 68.7 (11.4)	NM	NM
Castro 2014^[33]^	group 1: 244 group 2: 140	group 1: 78 (32) group 2: 42 (30)	group 1: 47.2 (12.9) group 2: 50.0 (11.5)	group 1: 0.54 (0.32) group 2: 0.19 (0.12)	group 1: 65.3 (15.3) group 2: 66.8 (15.1)	NM	NM
Nowak 2015^[34]^	72	25 (35)	36.3 (6.8)	0.213 (0.393)	58.1	NM	NM
Park HS 2016^[35]^	77	29 (38)	53.4 (11.5)	0.72 (0.87)	67.8 (14.4)	NM	NM
Bleecker E R 2016^[36]^	797	270(34)	48.9(14)	0.34(0.52)	56.8(14.4)	14.9	NM
FitzGerald J M 2016^[37]^	866	323(37)	49.5(14)	0.39(0.42)	58.4(14.9)	16.3	NM

*Data on all patients who received anti-interleukin 5, and all data are n (%) or mean (SD), unless otherwise stated.

^#^Geometric mean on loge scale.

^&^geometric means±log10 SD.

NM: Not Mentioned

### Primary outcomes

#### Lung function

**Fig 2:**
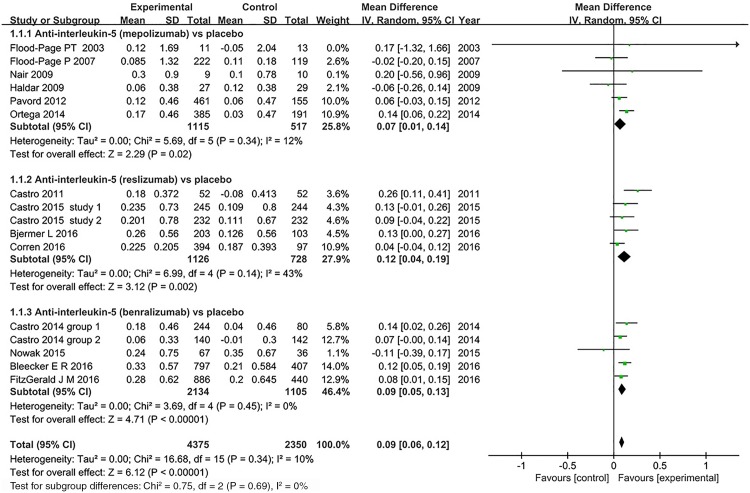
The effect of anti-interleukin 5 versus placebo on FEV_1_. CI = confidence interval; FEV_1_ = forced expiratory volume in 1 second; SD = standard derivation; IV = Inverse Variance.

***FEV***_***1***_. Fourteen studies assessed FEV_1_ responsiveness to anti-interleukin 5 treatment [19,21–24,26,28–31,33,34,36,37]. Six studies reported significant improvements in FEV1 between mepolizumab, reslizumab, and benralizumab treatments and placebo, while the remaining studies reported no effect on FEV1. Fig 2 showed that reslizumab was more effective than other two anti-interleukin 5 monoclonal antibodies in improving FEV_1_ (MD = 0.12, 95% CI: 0.04–0.19, *P* = 0.002), and the pooled data analysis revealed a slight improvement (MD = 0.09, 95% CI: 0.06–0.12, *P*<0.001). There was minimal heterogeneity (*I*^*2*^ = 10%, *P* = 0.34, 95% CI -53% to 47%, $H_{M}^{2}=0.10$).

#### FEV_1_% of predicted value

**Fig 3:**
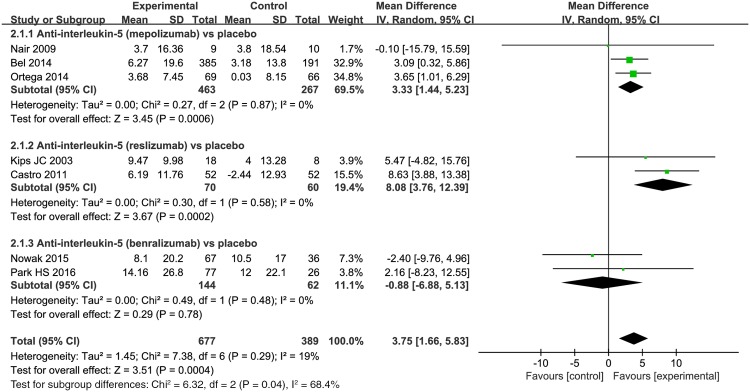
The effect of anti-interleukin 5 versus placebo on FEV_1_% of predicted value.

Seven trials using three different anti-interleukin 5 antibodies reported FEV_1_% of predicted value [23,25–28,34,35]. Overall, anti-interleukin 5 treatment was associated with modestly improved in FEV_1_% of predicted value compared to placebo (MD = 3.75, 95% CI 1.66–5.83, *P* = 0.0004) (Fig 3), and heterogeneity was not statistically significant (*I*^*2*^ = 19%, P = 0.29, 95% CI 0% to 62%, $H_{M}^{2}=0.23$). When looking at subgroups, there were no differences by benralizumab (MD = -0.88, 95% CI -6.88–5.13, *P* = 0.78).

#### Peak expiratory flow (PEF) and Provocative concentration of histamine (histamine PC_20_)

**Fig 4:**
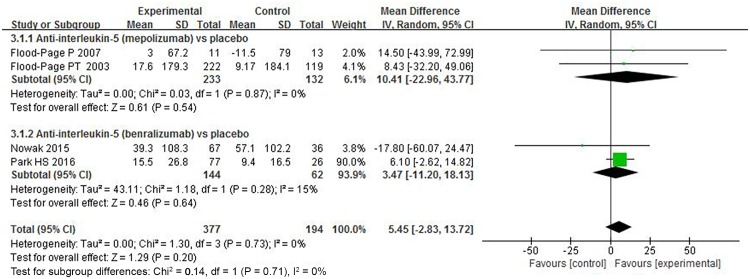
The effects of anti–interleukin-5 on PEF (L/min).

**Fig 5:**
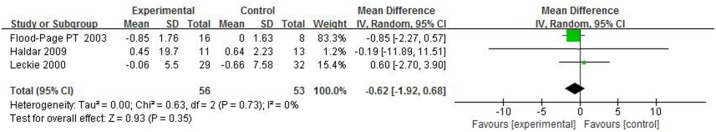
The effects of anti–interleukin-5 on histamine PC_20_ (mg/ml).

Four trials depicted PEF change after anti-interleukin 5 treatment [19,21,34,35], and only three about mepolizumab studies reported the results in histamine PC_20_ [18,19,22]. Results from the pooled data illustrated that anti-interleukin 5 could not significantly improve PEF (MD = 5.45, 95% CI: -2.83–13.72, *P* = 0.2) (Fig 4) or PC_20_ (MD = -0.62, 95% CI: -1.92–0.68, *P* = 0.35) (Fig 5). Studies were highly homogeneous (*I*^*2*^ = 0%, *P* = 0.73, 95% CI 0% to 84%, $H_{M}^{2}=0$; *I*^*2*^ = 0%, *P* = 0.73, 95% CI 0% to 89%, $H_{M}^{2}=0$). Our confidence in these results is low due to the wide CI.

#### Asthma Quality of Life Questionnaire (AQLQ) score

**Fig 6:**
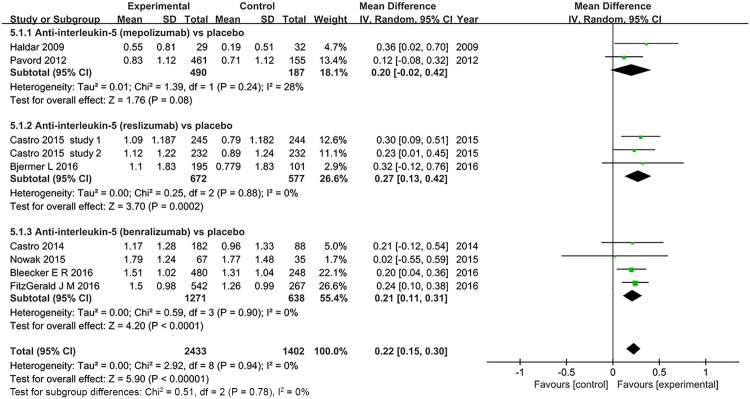
The effects of anti-interleukin 5 on Asthma Quality of Life Questionnaire (AQLQ).

Eight trials of three different anti-interleukin 5 antibodies reported AQLQ scores [22,24,29,31,33,34,36,37]. Pooled analysis showed that anti-interleukin 5 treatment was associated with significantly improved AQLQ score (MD = 0.22, 95% CI 0.15–0.30, *P*<0.001), with no significant heterogeneity (*I*^*2*^ = 0%, *P* = 0.94, 95% CI 0% to 29%, $H_{M}^{2}=-0.64$) (Fig 6). Among subgroups, AQLQ scores improved only in the trials involving reslizumab and benralizumab treatment trials (MD = 0.27, 95% CI 0.13–0.42, *P* = 0.0002; MD = 0.21, 95% CI 0.11–0.31, *P*<0.001), but not mepolizumab (*P* = 0.08).

#### Asthma exacerbations

**Fig 7:**
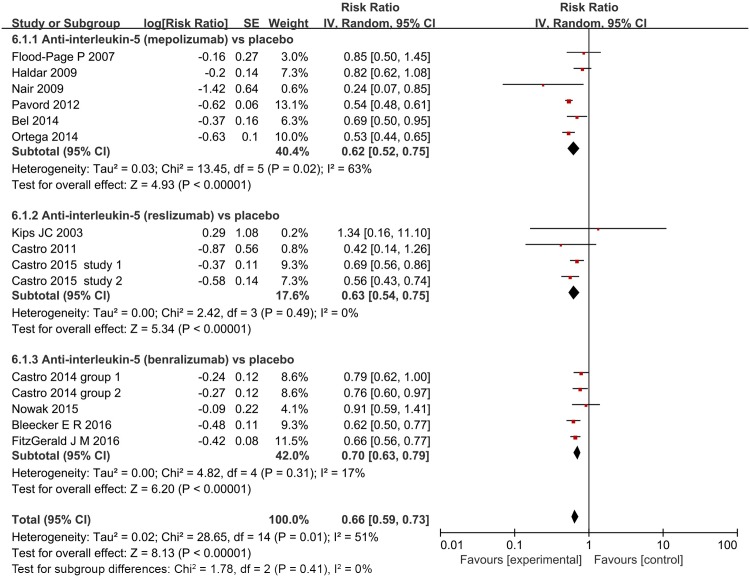
The effect of anti-interleukin 5 versus placebo on exacerbation. IV = Inverse Variance.

Thirteen studies (6,072 participants) reported on asthma exacerbations [21–29,33,34,36,37]. Table 1 summarizes their definitions for asthma exacerbation. Although these definitions varied, all 13 studies defined exacerbation based on increased corticosteroids or albuterol dose to control symptoms and/or the need for asthma-related emergency treatment/hospitalization. Fig 7 showed that anti-interleukin 5 monoclonal therapies were associated with a significant reduction in asthmatic exacerbation compared with placebo (RR = 0.66, 95% CI, 0.59–0.73, *P*<0.001), but the reporting was significantly heterogeneous (*I*^*2*^ = 51%, *P*<0.001, 95% CI 12% to 73%, $H_{M}^{2}=1.05$).

### Secondary outcomes

#### Blood and sputum eosinophils

18 trials included blood eosinophil analysis and six trials compared sputum eosinophil levels between anti-interleukin 5 treatment and placebo [18,22–24,28,32]. As the data were reported inconsistently (data were shown as median [range], mean [SD] or geometric mean [log10 SE]), we did not obtain a synthesized analysis of the outcomes. However, from all the results reported, a similar outcome was identified that anti-interleukin 5 significantly decreased blood and sputum eosinophils compared with placebo (S2 Table).

#### SABA rescue use

**Fig 8:**
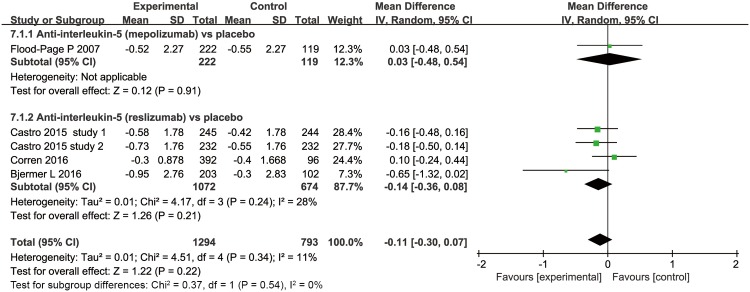
The effects of anti-interleukin-5 on SABA rescue use.

Four trials evaluated the effect of anti-interleukin 5 antibodies on SABA use (Fig 8) [21,29–31]. Analyses of these studies showed a non-significant decrease in the anti–interleukin 5 group compared with the placebo group (MD = -0.11, 95% CI -0.3–0.07, *P* = 0.22), with low heterogeneity (*I*^*2*^ = 11%, *P* = 0.34, 95% CI 0% to 54%, $H_{M}^{2}=0.13$) among the studies.

#### Adverse events

**Fig 9:**
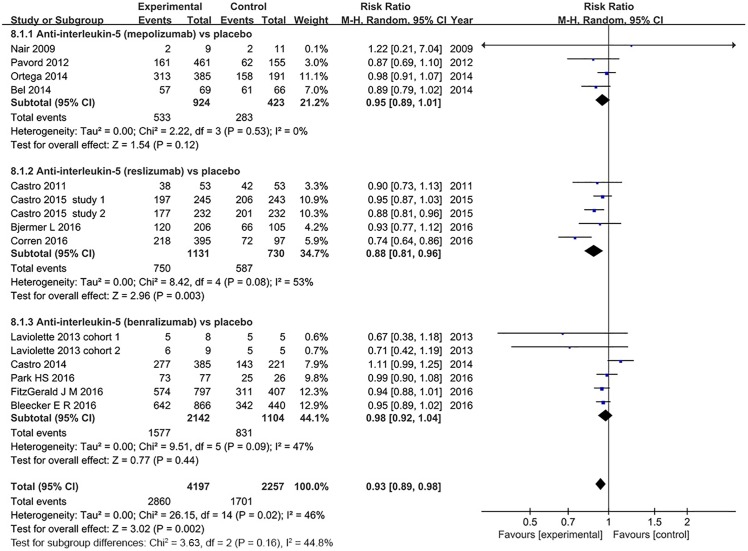
The effect of anti-interleukin 5 versus placebo on adverse events.

13 studies reported adverse events, and anti-interleukin 5 therapy was well tolerated [23–26,28–33,35–37]. The pooled RR was 0.93 (95% CI: 0.89–0.98), which showed the lower adverse events incidence were slightly in the anti-interleukin 5 group (*P* = 0.002), with modest heterogeneity (*I*^*2*^ = 46%, *P* = 0.02, 95% CI 2.3% to 71%, $H_{M}^{2}=0.87$) (Fig 9). However, sensitivity analysis that excluded two studies which included patients with non-eosinophilic asthma revealed no heterogeneity (*I*^*2*^ = 0%, *P* = 0.75, 95% CI 0% to 48%, $H_{M}^{2}=-0.3$) [30,33]. Therefore, the heterogeneity can be explained by the varied participant types. In subgroup analysis, however, only treatment with reslizumab was associated with a trend of lower adverse events incidence (RR = 0.88, 95% CI: 0.81–0.96, *P* = 0.003), while no significant differences were found in both mepolizumab (RR = 0.95, 95% CI: 0.89–1.01, *P* = 0.12) and benralizumab treatment groups (RR = 0.98, 95% CI: 0.92–1.04 *P* = 0.44).

### Risk of bias

**Fig 10:**
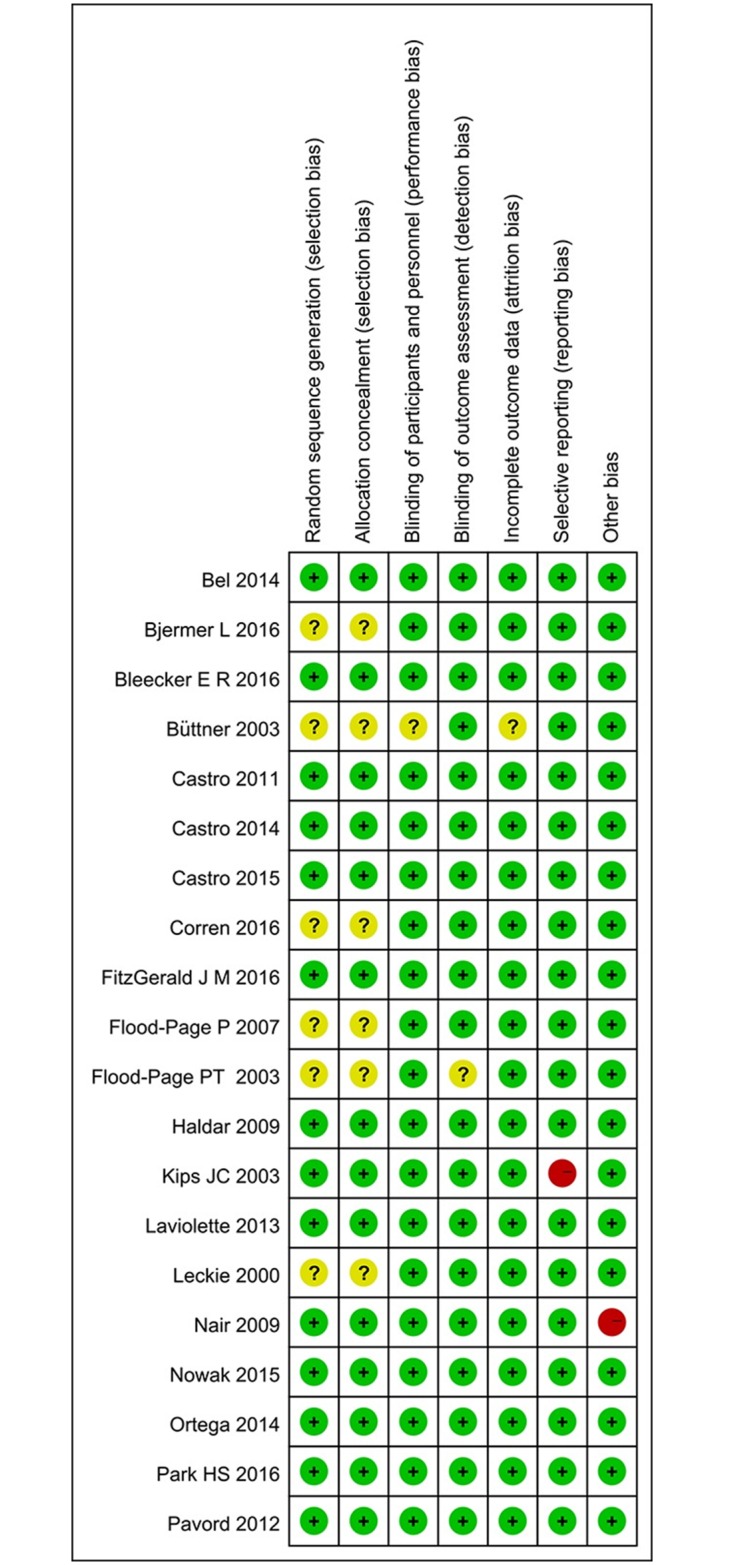
Risk of bias summary.

Fig 10 summarizes the methodological domain assessments for each included study. Most trials had low risk of bias across the six domains. The allocation sequence was adequately generated and concealed in fourteen trials, [22–29,32–37]. The randomization techniques included computer generated randomization codes and minimization. The remaining trials did not report the method used, and we were unable to obtain this information. All but one study was described as double-blinded [20]. Almost all RCTs reported complete outcome data, only one trial reported on attrition insufficiently [27].

### Subgroup analyses and sensitivity analysis

To clarify the heterogeneity or identify the optimal patients for this treatment, subgroup analyses were carried out for asthma exacerbations and FEV_1_ (Table 5). The studies were stratified according to effects model, asthma severity, asthma types, sample size, drug administration dosage, follow-up duration and published year. Subgroup analyses showed the efficacy of anti-interleukin 5 on asthma exacerbations were only influenced by asthma severity. Most subgroups showed significantly reduced exacerbations risk. Single dose anti-interleukin 5 in two studies showed no significant differences in exacerbation rates. However, the subgroup results should be interpreted with caution because of the limited sample size and potential bias inherent to subgroup analysis. The meta-analysis findings remained stable with multicenter trials. In addition, excluding the results of any single study did not alter the overall findings. Furthermore, based on the subgroups analysis, we could know that anti-interleukin 5 can significantly improve the FEV_1_ of severe asthma (MD = 0.11, *P*<0.001) and eosinophilic asthma (MD = 0.11, *P* = 0.001). This further confirmed that the severe eosinophilic asthma patients are the optimal patients for anti-interleukin 5 treatment.

**Table 5:** Subgroup analysis and sensitivity analyses of asthma exacerbation and FEV_1_ in RCTs.

Stratification	asthma exacerbation				FEV_1_			
	No. of Patients (Studies)	RR(95% CI)	*P* Value	*I*^*2*^, %	No. of Patients (Studies)	MD(95% CI)	*P* Value	*I*^*2*^, %
**Subgroup analysis**								
**Effects model**								
random-effects model	6072(13)	0.66(0.59–0.73)	<0.001	51	6725(14)	0.09(0.06–0.12)	<0.001	10
fixed effects model	6072(13)	0.63(0.59–0.67)	<0.001	51	6725(14)	0.09(0.06–0.12)	<0.001	10
**Asthma severity**								
mild or moderate asthma	362(1)	0.85(0.51–1.43)	0.55	…	365(2)	-0.02(-0.2–0.15)	0.8	0
severe asthma	4090(8)	0.59(0.53–0.65)	<0.001	23	3901(7)	0.11(0.07–0.14)	<0.001	35
mixed asthma	1620(4)	0.73(0.65–0.82)	<0.001	18	2459(5)	0.08(0.04–0.12)	<0.001	0
**Asthma types**								
eosinophilic asthma	3117(7)*	0.64(0.56–0.74)	<0.001	65	3002(6)*	0.11(0.05–0.17)	<0.001	46
mon-eosinophilic asthma	282(1)	0.76(0.60–0.97)	0.02	…	773(2)	0.06(0.00–0.11)	0.05	0
mixed asthma	2673(6)	0.66(0.57–0.77)	<0.001	24	2950(7)	0.10(0.05–0.15)	<0.001	0
**No. of subjects**								
<100	107(3)	0.63(0.28–1.45)	0.28	46	99(3)	-0.04(-0.23–0.15)	0.68	0
≥100	5965(10)	0.65(0.59–0.72)	<0.001	51	6626(11)	0.09(0.06–0.13)	<0.001	16
**Follow-up**								
<50 weeks	2530(8)	0.64(0.54–0.76)	<0.001	33	3168(9)	0.10(0.05–0.16)	<0.001	34
≥50 weeks	3542(5)	0.67(0.59–0.76)	<0.001	67	3557(5)	0.08(0.04–0.12)	<0.001	0
**Intervention dosage**								
single dose	134(2)	0.93(0.61–1.42)	0.73	0	103(1)	-0.11(-0.39–0.17)	0.45	…
multiple doses	5938(11)	0.65(0.57–0.74)	<0.001	58	6622(13)	0.09(0.07–0.12)	<0.001	5
**Year**								
published year ≤2011	575(5)	0.73(0.52–1.02)	0.07	21	544(5)	0.08(-0.01–0.25)	0.4	54
published year >2011	5497(8)	0.64(0.58–0.71)	<0.001	56	6181(9)	0.09(0.06–0.12)	<0.001	0
**Sensitivity analysis**								
Non-multicenter	5991(11)	0.65(0.59–0.72)	<0.001	47	6626(11)	0.09(0.06–0.13)	<0.001	16
One-study-out method	…	…	From 0.65 (0.58–0.71) to 0.68 (0.61–0.75)	…		…		

*The Castro 2015 inclued two groups, group 1 for eosinophilic asthma, group 2 for non-eosinophilic asthma.

### Publication bias

**Fig 11:**
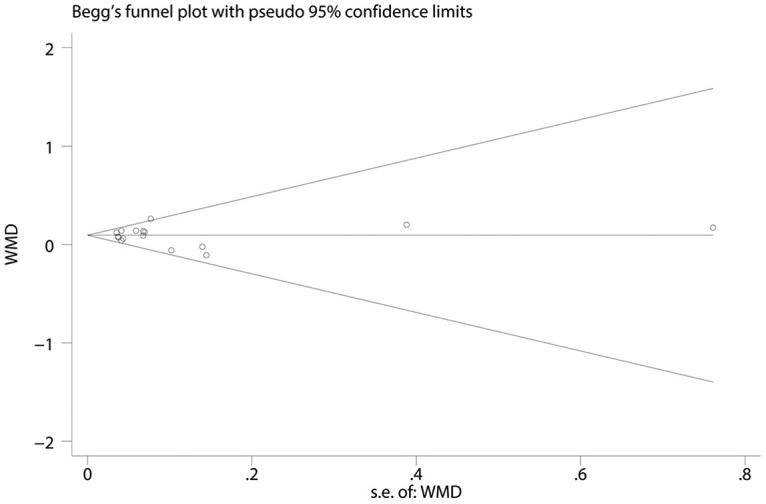
Begg’s funnel plot evaluated the effect of anti-interleukin-5 on FEV_1_.

Publication bias was assessed using Begg’s funnel plot and Egger’s test. Begg’s funnel plot of the 14 studies evaluated the effect of anti-interleukin 5 on FEV1 and the Egger’s test suggested no publication bias (*P* = 0.78, Fig 11). And also no publication bias was detected by Egger’s test for other outcomes analysis (all *P*>0.05). However, we could not fully exclude publication bias in four outcomes (FEV_1_%, PEF, histamine PC20, SABA rescue use); we could not evaluate the potential risk of publication bias, since these tests have very low power in meta-analysis.

## Discussion

We identified 20 RCTs investigating the effect of anti-interleukin 5 monoclonal antibodies in patients with asthma. The results suggest that anti-interleukin 5 therapy was well tolerated and could significantly improve AQLQ score, FEV_1_, FEV_1_% of predicted value, and decrease asthmatic exacerbation, blood and sputum eosinophil levels, but yielded no effects in PEF, PC_20_, SABA rescue use. Additionally, reslizumab seems to be safer and more effective than the other two drugs based on all outcomes. However, since varied baseline of patients among studies, it is not possible to draw a firm conclusion. Different from previous systematic reviews that only included studies of on mepolizumab [38, 39], we included trials about mepolizumab and other two anti-interleukin-5 antibodies-reslizumab and benralizumab. Additionally, the results should be interpreted with caution due to with the relatively small sample sizes and small number of included trials. Therefore, our results may be more believable. In contrast to previous systematic reviews, we found that anti-interleukin-5 treatment slightly increased FEV_1_ and FEV_1_% of predicted value. But the clinical relevance of this finding to patients may not be clinically important because of the modest improvement. Only three or four studies reported detailed data, therefore we could not draw exact conclusions for these two parameters due to the insufficient data. Previous two systematic reviews failed to show a significant effect in FEV_1_, likely due to small number of trials analyzed [38, 39]. Liu et al [39] converted and pooled continuous variable data such as blood and sputum eosinophils. To reduce the possible bias resulting from data conversion, we only obtained qualitative descriptions with estimations of the two outcomes. Besides, when studies with multiple intervention groups, Liu et al [39] only selected one pair of interventions and exclude the others which are not generally recommended by Cochrane handbook. Our meta-analysis found that there was a significant improvement in AQLQ score, which is consistent with previous two meta-analyses. However, as the mean change in AQLQ score is less than the clinical minimally important difference of 0.5 units, the clinical relevance of this finding may not be clinically important to patients [40]. Asthma exacerbations are associated with substantial morbidity and mortality [41]. Decreasing the asthma exacerbations rate is a key goal in asthma management. Our meta-analysis showed a significant reduction in exacerbation rates. The clinical relevance of this finding to patients may be clinically important. The inconsistency of the results between asthma exacerbations and rescue use of SABA might due to the next two reasons: 1) the slight improvement in lung functions; 2) most exacerbations in trials were predominantly those that would generally be judged severe on the basis of a need for systemic corticosteroid or requiring admission or visit to emergency. This systematic review also has limitations. First, we aimed to identify the overall effect of anti-interleukin-5 therapy on asthma, the asthma severity and baseline asthma therapy varied among studies (Table 2), so the population examined in this review was too heterogeneous to draw any conclusions about the general asthma population. Further research is needed to clarify which subgroups of patients with asthma can benefit from this treatment. Second, in accordance with the Cochrane handbook, we combined two or three intervention groups into a single intervention group regardless of different intervention dosage and administration routine. This made identifying the optimal dose and regimen for treating asthma difficult. Thirdly, although these studies shared many common issues, there were also substantial subgroup and study heterogeneities. Moreover, there also was significant heterogeneity among studies evaluating asthma exacerbation and adverse events; although we used a random-effects model to account for this, the correction is only partial. As for PEF, histamine PC20 and SABA rescue use, given the small number of studies being meta-analysed, it is difficult to detect heterogeneity and accurately estimate it [42]. Lastly, Ortega et al [43] re-examined baseline blood eosinophil counts from previous two studies [24,26] on mepolizumab, they found that the use of the baseline at a threshold of at least 150 cells/μL can be a reliable and simple biomarker for patient selection associated with positive clinical responses to mepolizumab treatment. However, due to the lack of individual patient data among all studies, we failed to further analysis the relationship between blood eosinophil counts≥150 cells/μL at baseline and outcomes of mepolizumab, reslizumab and benralizumab treatment.

## Conclusions

Our study indicates that anti-interleukin-5 therapy is safe and may reduce asthma exacerbation risk, slightly improve FEV_1_, FEV_1_%, and quality of life; and decrease blood and sputum eosinophil levels, although PEF, PC_20_ were not improved or SABA rescue use reduced. Anti-interleukin-5 therapy may therefore be beneficial as adjunct asthma therapy, particularly in severe and eosinophilic asthma. Further trials are necessary to determine the most effective asthma treatment drug and studies need to be performed that distinguish which patients will respond to particular antibodies, both within and between classes (i.e., who will respond to mepolizumab vs. benralizumab or? reslizumab vs. benralizumab?).

## Supporting Information

S1 Table Search strategies. (DOCX)

S2 Table Secondary efficacy outcomes of included RCTs. (DOCX)

S3 Table The data of all outcomes in all RCTs. (DOCX)

S4 Table PRISMA 2009 Checklist. (DOCX)

## References

[1] The Global Asthma Report 2014. Auckland, New Zealand: Global Asthma Network, 2014. Available: http://www.globalasthmareport.org/resources/Global_Asthma_Report_2014.pdf.

[2] Bateman ED, Hurd SS, Barnes PJ, Bousquet J, Drazen JM, FitzGerald M, et al Global strategy for asthma management and prevention: GINA executive summary. Eur Respir J. 2008;31(1):143–78. 10.1183/09031936.00138707 . 18166595

[3] Masoli M, Fabian D, Holt S, Beasley R, Global Initiative for Asthma P. The global burden of asthma: executive summary of the GINA Dissemination Committee report. Allergy. 2004;59(5):469–78. 10.1111/j.1398-9995.2004.00526.x . 15080825

[4] McIvor RA. Emerging therapeutic options for the treatment of patients with symptomatic asthma. Ann Allergy Asthma Immunol. 2015;115(4):265–71.e5 10.1016/j.anai.2015.07.011 26254973

[5] Torrego A, Sola I, Munoz AM, Roque IFM, Yepes-Nunez JJ, Alonso-Coello P, et al Bronchial thermoplasty for moderate or severe persistent asthma in adults. Cochrane Database Syst Rev. 2014;(3):CD009910 10.1002/14651858.CD009910.pub2 . 24585221 PMC6986472

[6] Chung KF. Targeting the interleukin pathway in the treatment of asthma. The Lancet. 2015;386(9998):1086–96. 10.1016/S0140-6736(15)00157-9 26383000

[7] Mitchell PD, El-Gammal AI, O'Byrne PM. Emerging monoclonal antibodies as targeted innovative therapeutic approaches to asthma. Clin Pharmacol Ther. 2016;99(1):38–48. 10.1002/cpt.284 26502193

[8] Cardet JC, Israel E. Update on reslizumab for eosinophilic asthma. Expert Opin Biol Ther. 2015;15(10):1531–9. 10.1517/14712598.2015.1090972 26372797 PMC5161345

[9] Yamaguchi Y, Suda T, Suda J, Eguchi M, Miura Y, Harada N, et al Purified interleukin 5 supports the terminal differentiation and proliferation of murine eosinophilic precursors. J Exp Med. 1988;167(1):43–56. 3257253 10.1084/jem.167.1.43 PMC2188821

[10] Garcia G, Taille C, Laveneziana P, Bourdin A, Chanez P, Humbert M. Anti-interleukin-5 therapy in severe asthma. Eur Respir Rev. 2013;22(129):251–7. 10.1183/09059180.00004013 23997052 PMC9487362

[11] Takatsu K, Nakajima H. IL-5 and eosinophilia. Curr Opin Immunol. 2008;20(3):288–94. 10.1016/j.coi.2008.04.001 18511250

[12] Patterson MF, Borish L, Kennedy JL. The past, present, and future of monoclonal antibodies to IL-5 and eosinophilic asthma: A review. J Asthma Allergy. 2015;8:125–34. 10.2147/JAA.S74178 26604804 PMC4639549

[13] Durham AL, Caramori G, Chung KF, Adcock IM. Targeted anti-inflammatory therapeutics in asthma and chronic obstructive lung disease. Transl Res. 2016;167(1):192–203. 10.1016/j.trsl.2015.08.004 26334389 PMC4728194

[14] Higgins J. Green S. Cochrane handbook for systematic reviews of interventions version 5.1. 0. The Cochrane Collaboration, 2011. 2013.

[15] Mittlbock M, Heinzl H. A simulation study comparing properties of heterogeneity measures in meta-analyses. Stat Med. 2006;25(24):4321–33. 10.1002/sim.2692 . 16991104

[16] Ioannidis JP, Patsopoulos NA, Evangelou E. Uncertainty in heterogeneity estimates in meta-analyses. BMJ (Clinical researched). 2007;335(7626):914–6. 10.1136/bmj.39343.408449.80 17974687 PMC2048840

[17] Higgins JP, Thompson SG. Quantifying heterogeneity in a meta-analysis. Stat Med. 2002;21(11):1539–58. 10.1002/sim.1186 . 12111919

[18] Leckie MJ, Ten Brinke A, Khan J, Diamant Z, O'Connor BJ, Walls CM, et al Effects of an interleukin-5 blocking monoclonal antibody on eosinophils, airway hyper-responsiveness, and the late asthmatic response. The Lancet. 2000;356(9248):2144–8. 10.1016/S0140-6736(00)03496-6 11191542

[19] Flood-Page P, Menzies-Gow A, Phipps S, Ying S, Wangoo A, Ludwig MS, et al Anti-IL-5 treatment reduces deposition of ECM proteins in the bronchial subepithelial basement membrane of mild atopic asthmatics. Am J Respir Crit Care Med. 2003;112(7):1029–36. 10.1172/JCI17974 . 14523040 PMC198522

[20] Büttner C, Lun A, Splettstoesser T, Kunkel G, Renz H. Monoclonal anti-interleukin-5 treatment suppresses eosinophil but not T-cell functions. Eur Respir J. 2003; 21(5):799–803. 10.1183/09031936.03.00027302 12765424

[21] Flood-Page P, Swenson C, Faiferman I, Matthews J, Williams M, Brannick L, et al A study to evaluate safety and efficacy of mepolizumab in patients with moderate persistent asthma. Am J Respir Crit Care Med. 2007;176(11):1062–71. 10.1164/rccm.200701-085OC 17872493

[22] Haldar P, Brightling CE, Hargadon B, Gupta S, Monteiro W, Sousa A, et al Mepolizumab and exacerbations of refractory eosinophilic asthma. N Engl J Med. 2009;360(10):973–84. 10.1056/NEJMoa0808991 19264686 PMC3992367

[23] Nair P, Pizzichini MMM, Kjarsgaard M, Inman MD, Efthimiadis A, Pizzichini E, et al Mepolizumab for prednisone-dependent asthma with sputum eosinophilia. N Engl J Med. 2009;360(10):985–93. 10.1056/NEJMoa0805435 19264687

[24] Pavord ID, Korn S, Howarth P, Bleecker ER, Buhl R, Keene ON, et al Mepolizumab for severe eosinophilic asthma (DREAM): A multicentre, double-blind, placebo-controlled trial. The Lancet. 2012;380(9842):651–9. 10.1016/S0140-6736(12)60988-X 22901886

[25] Bel EH, Wenzel SE, Thompson PJ, Prazma CM, Keene ON, Yancey SW, et al Oral glucocorticoid-sparing effect of mepolizumab in eosinophilic asthma. N Engl J Med. 2014;371(13):1189–97. 10.1056/NEJMoa1403291 25199060

[26] Ortega HG, Liu MC, Pavord ID, Brusselle GG, FitzGerald JM, Chetta A, et al Mepolizumab treatment in patients with severe eosinophilic asthma. N Engl J Med. 2014;371(13):1198–207. 10.1056/NEJMoa1403290 . 25199059

[27] Kips JC, O'Connor BJ, Langley SJ, Woodcock A, Kerstjens HA, Postma DS, et al Effect of SCH55700, a humanized anti-human interleukin-5 antibody, in severe persistent asthma: a pilot study. Am J Respir Crit Care Med. 2003;167(12):1655–9. Epub 2003/03/22. 10.1164/rccm.200206-525OC . 12649124

[28] Castro M, Mathur S, Hargreave F, Boulet LP, Xie F, Young J, et al Reslizumab for poorly controlled, eosinophilic asthma: a randomized, placebo-controlled study. Am J Respir Crit Care Med. 2011;184(10):1125–32. Epub 2011/08/20. 10.1164/rccm.201103-0396OC . 21852542

[29] Castro M, Zangrilli J, Wechsler ME, Bateman ED, Brusselle GG, Bardin P. Reslizumab for inadequately controlled asthma with elevated blood eosinophil counts: results from two multicentre, parallel, double-blind, randomised, placebo-controlled, phase 3 trials. Lancet Respir Med. 2015; 3(5):355–66. 10.1016/S2213-2600(15)00042-9 25736990

[30] Corren J, Weinstein S, Janka L, Zangrilli J, Garin M. Phase 3 Study of Reslizumab in Patients with Poorly Controlled Asthma: Effects Across a Broad Range of Eosinophil Counts. Chest. 2016; 45715–6. 10.1016/j.chest.2016.03.018 . 27018175

[31] Bjermer L, Lemiere C, Maspero J, Weiss S, Zangrilli J, Germinaro M. Reslizumab for Inadequately Controlled Asthma with Elevated Blood Eosinophil Levels: a Randomized Phase 3 Study. Chest. 2016; 47551–3. 10.1016/j.chest.2016.03.032 . 27056586

[32] Laviolette M, Gossage DL, Gauvreau G, Leigh R, Olivenstein R, Katial R, et al Effects of benralizumab on airway eosinophils in asthmatic patients with sputum eosinophilia. J Allergy Clin Immunol. 2013;132(5):1086–96.e5 10.1016/j.jaci.2013.05.020 23866823 PMC4172321

[33] Castro M, Wenzel SE, Bleecker ER, Pizzichini E, Kuna P, Busse WW, et al Benralizumab, an anti-interleukin 5 receptor alpha monoclonal antibody, versus placebo for uncontrolled eosinophilic asthma: A phase 2b randomised dose-ranging study. Lancet Respir Med. 2014; 2(11):878–90. 10.1016/S2213-2600(14)70201-2 . 25306557

[34] Nowak RM, Parker JM, Silverman RA, Rowe BH, Smithline H, Khan F. A randomized trial of benralizumab, an antiinterleukin 5 receptor alpha monoclonal antibody, after acute asthma. Am J Emerg Med. 2015; 33(1):14–20. 10.1016/j.ajem.2014.09.036 . 25445859

[35] Park HS, Kim MK, Imai N, Nakanishi T, Adachi M, Ohta K, et al A phase 2a study of benralizumab for patients with eosinophilic asthma in South Korea and Japan. Int Arch Allergy Immunol. 2016;169(3):135–45. 10.1159/000444799 27097165

[36] Bleecker ER, FitzGerald JM, Chanez P, Papi A, Weinstein SF, Barker P, et al Efficacy and safety of benralizumab for patients with severe asthma uncontrolled with high-dosage inhaled corticosteroids and long-acting beta2-agonists (SIROCCO): a randomised, multicentre, placebo-controlled phase 3 trial. Lancet. 2016 10.1016/S0140-6736(16)31324-1 . 27609408

[37] FitzGerald JM, Bleecker ER, Nair P, Korn S, Ohta K, Lommatzsch M, et al Benralizumab, an anti-interleukin-5 receptor alpha monoclonal antibody, as add-on treatment for patients with severe, uncontrolled, eosinophilic asthma (CALIMA): a randomised, double-blind, placebo-controlled phase 3 trial. Lancet. 2016 10.1016/S0140-6736(16)31322-8 . 27609406

[38] Powell C, Milan SJ, Dwan K, Bax L, Walters N. Mepolizumab versus placebo for asthma. Cochrane Database Syst Rev. 2015;7:CD010834 10.1002/14651858.CD010834.pub2 . 26214266

[39] Liu Y, Zhang S, Li DW, Jiang SJ. Efficacy of Anti-Interleukin-5 Therapy with Mepolizumab in Patients with Asthma: A Meta-Analysis of Randomized Placebo-Controlled Trials. PloS one. 2013; 8(3):e59872 10.1371/journal.pone.0059872 . 23544105 PMC3609729

[40] Juniper EF, Guyatt GH, Willan A, Griffith LE. Determining a minimal important change in a disease-specific Quality of Life Questionnaire. J Clin Epidemiol. 1994;47(1):81–7. 10.1016/0895-4356(94)90036-1 . 8283197

[41] Global Initiative for Asthma. Global strategy for asthma management and prevention, 2016. Available: http://ginasthma.org/wp-content/uploads/2016/04/GINA-2016-main-report_tracked.pdf.

[42] Kontopantelis E, Springate DA, Reeves D. A re-analysis of the Cochrane Library data: the dangers of unobserved heterogeneity in meta-analyses. PloS one. 2013;8(7):e69930 10.1371/journal.pone.0069930 23922860 PMC3724681

[43] Ortega HG, Yancey SW, Mayer B, Gunsoy NB, Keene ON, Bleecker ER, et al Severe eosinophilic asthma treated with mepolizumab stratified by baseline eosinophil thresholds: a secondary analysis of the DREAM and MENSA studies. Lancet Respir Med. 2016;4(7):549–56. 10.1016/S2213-2600(16)30031-5 . 27177493

