# Supplementary material for: Efficacy and Safety of Anti-Interleukin-5 Therapy in Patients with Asthma: A Systematic Review and Meta-Analysis
Source: PLoS One. 2016 Nov 22;11(11):e0166833. doi: 10.1371/journal.pone.0166833 (PMC5119789; doi:10.1371/journal.pone.0166833)
Supplement: S1 Table — (DOCX) [file pone.0166833.s001.docx]

**S1 Table. Search strategies**

**Source: Pubmed**

**Searched on:** October 10, 16

**Results:** 2019

| **Search** | **Query** | **Results** |
| --- | --- | --- |
| #1 | "mepolizumab" [Supplementary Concept] | [132](http://www.ncbi.nlm.nih.gov/pubmed/?cmd=HistorySearch&querykey=2) |
| #2 | Search ((mepolizumab) OR SB-240563) OR SB240563 | 250 |
| #3 | #1 OR #2 | [250](http://www.ncbi.nlm.nih.gov/pubmed/?cmd=HistorySearch&querykey=4) |
| #4 | " reslizumab " [Supplementary Concept] | [22](http://www.ncbi.nlm.nih.gov/pubmed/?cmd=HistorySearch&querykey=6) |
| #5 | Search ((reslizumab) OR SCH55700) | [71](http://www.ncbi.nlm.nih.gov/pubmed/?cmd=HistorySearch&querykey=7) |
| #6 | #4 OR #5 | [71](http://www.ncbi.nlm.nih.gov/pubmed/?cmd=HistorySearch&querykey=8) |
| #7 | " benralizumab " [Supplementary Concept] | [13](http://www.ncbi.nlm.nih.gov/pubmed/?cmd=HistorySearch&querykey=13) |
| #8 | Search (benralizumab) OR MEDI-563 | [43](http://www.ncbi.nlm.nih.gov/pubmed/?cmd=HistorySearch&querykey=14) |
| #9 | #7 OR #8 | [43](http://www.ncbi.nlm.nih.gov/pubmed/?cmd=HistorySearch&querykey=15) |
| #10 | Search (anti–IL-5) OR anti–interleukin-5 | [485](http://www.ncbi.nlm.nih.gov/pubmed/?cmd=HistorySearch&querykey=17) |
| #11 | Search antibodies, monoclonal, humanized[MeSH Terms] | 31873 |
| #12 | #10 OR #11 | 32291 |
| #13 | Search asthma[MeSH Terms] | 113632 |
| #14 | Search asthma[Title/Abstract] | 125232 |
| #15  #16  #17 | #13 OR #14  #3 OR #6 OR #12  #15 AND #16 Filters: Humans | 150445  32391  2019 |

**Source: EMBASE**

**Searched on:** October 10, 16

**Results:** 893

| **Search** | **Query** | **Results** |
| --- | --- | --- |
| #1 | exp asthma/ | 174119 |
| #2 | asthma.ab,ti. | 136297 |
| #3 | #1 OR #2 | 185887 |
| #4 | exp mepolizumab/ | 1209 |
| #5 | mepolizumab.ab,ti. | 309 |
| #6 | #4 OR #5 | 1224 |
| #7 | exp reslizumab/ | 346 |
| #8 | reslizumab.ab,ti. | 71 |
| #9  #10  #11  #12  #13  #14 | #7 OR #8  exp benralizumab/  benralizumab.ab,ti.  #10 OR #11  #6 OR #9 OR #12  #3 AND #13 limit to human | 351  203  51  203  1343  893 |

**Source: Cochrane Central Register of Controlled Trials**

**Searched on:** October 10, 16

**Results:** 135

| **Search** | **Query** | **Results** |
| --- | --- | --- |
| #1 | [asthma][Mesh] | 9880 |
| #2 | asthma:ti,ab,kw | 23988 |
| #3 | #1 OR #2 | 23988 |
| #4 | mepolizumab:ti,ab,kw OR SB-240563:ti,ab,kw OR SB240563:ti,ab,kw | 91 |
| #5 | reslizumab:ti,ab,kw OR SCH55700:ti,ab,kw | 29 |
| #6 | benralizumab:ti,ab,kw OR MEDI-563:ti,ab,kw | 36 |
| #7 | anti–IL-5 :ti,ab,kw OR anti–interleukin-5:ti,ab,kw | 59 |
| #8 | #4 OR #5 OR #6 OR #7 | 154 |
| #9 | #2 AND #8 | 135 |
|  |  |  |
