# Supplementary material for: Efficacy and Safety of Anti-Interleukin-5 Therapy in Patients with Asthma: A Systematic Review and Meta-Analysis
Source: PLoS One. 2016 Nov 22;11(11):e0166833. doi: 10.1371/journal.pone.0166833 (PMC5119789; doi:10.1371/journal.pone.0166833)
Supplement: S2 Table — (DOCX) [file pone.0166833.s002.docx]

**S2 Table.** **Secondary efficacy outcomes of included RCTs**

| **Ref** | **Patients (n)** | **Intervertion** | **Baseline** | **Treatment endpoint** | **P Value vs Placebo** | **Conclusion** |
| --- | --- | --- | --- | --- | --- | --- |
| **Blood eosinophils(10^9^/L)** |  |  |  |  |  |  |
| Leckie 2000^18^ | 8 | Mepolizumab 2.5 mg/kg | 0.5 | 0.09 | 0.0002 | Mepolizumab at doses of 2.5 mg/kg and 10 mg/kg lowered the mean blood eosinophil count |
|  | 8 | Mepolizumab 10 mg/kg | 0.36 | 0.04 | <0.0001 |  |
|  | 8 | Placebo | 0.74 | 0.52 |  |  |
| Flood-Page PT 2003^19^ | 11 | Mepolizumab 750 mg | 0.27(0.1-1.2) | NA | 0.02 | Mepolizumab at doses of 750 mg significantly decreased the blood eosinophil count |
|  | 13 | Placebo | 0.4(0.1-0.6) | NA |  |  |
| Büttner 2003^20^ | 7 | Mepolizumab 250 mg | NA | NA |  | Treatment with mepolizumab resulted in a marked, rapid and sustained decrease of blood eosinophil |
|  | 5 | Mepolizumab 750 mg | NA | NA |  |  |
|  | 7 | Placebo | NA | NA |  |  |
| Flood-Page P 2007^21^ | 120 | Mepolizumab 250 mg | 0.346(0.22) | NA | <0.01 | Mepolizumab caused a significant decrease inblood eosinophils at both doses |
|  | 116 | Mepolizumab 750 mg | 0.359(0.28) | NA | <0.01 |  |
|  | 126 | Placebo | 0.39(0.359) |  |  |  |
| Haldar 2009^22^ | 29 | Mepolizumab 750 mg | 0.32(0.38)^*^ | 0.048^*^ | <0.001 | Mepolizumab therapy was associated with significant reductions in blood eosinophil counts |
|  | 32 | Placebo | 0.35(0.3)^*^ | 0.315^*^ |  |  |
| Nair 2009^23^ | 9 | Mepolizumab 750 mg | 0.6644(0.49) | 0.0763(0.0394) | 0.004 | A single infusion of mepolizumab was associated with a reduction in the number of bllod eosinophils |
|  | 11 | Placebo | 0.3521(0.25) | 1.224(1.383) |  |  |
| Pavord 2012^24^ | 153 | Mepolizumab 75 mg | 0.25 (0.95)^#^ | NA | <0.0001 | Mepolizumab treatment lowers blood eosinophil counts |
|  | 152 | Mepolizumab 250 mg | 0.23 (1.20)^#^ | NA | <0.0001 |  |
|  | 156 | Mepolizumab 750 mg | 0.25 (0.93)^#^ | NA | <0.0001 |  |
|  | 155 | Placebo | 0.28(1.01)^#^ |  |  |  |
| Bel 2014^25^ | 69 | Mepolizumab 100 mg | 0.25(1.245)^#^ | NA | <0.001 | As compared with Placebo, mepolizumab significantly reduced blood eosinophil counts |
|  | 66 | Placebo | 0.23(1.001)^#^ |  |  |  |
| Ortega 2014^26^ | 191 | Mepolizumab 75 mg | 0.28(0.987)^#^ |  |  | Mepolizumab therapy was associated with significant reductions in blood eosinophils |
|  | 194 | Mepolizumab 100 mg | 0.29(1.05)^#^ |  |  |  |
|  | 191 | Placebo | 0.32(0.938)^#^ |  |  |  |
| Kips JC 2003^27^ | 6 | Mepolizumab 0.3 mg/kg | 0.28(0.10) |  | NA | Mepolizumab at 1 mg/kg therapy was associated with significant reductions in blood eosinophils |
|  | 12 | Mepolizumab 1 mg/kg | 0.23(0.14) | 0.07(0.03) | 0.05 |  |
|  | 8 | Placebo | 0.45(0.45) |  |  |  |
| Castro 2011^28^ | 53 | Placebo | 0.5 (0.00-1.20) | 0.00(–0.80-0.80) |  |  |
| Castro 2015 study 1^29^ | 245 | Reslizumab 3.0 mg/kg | 0.696 (0.768) | 0.114 | <0.0001 | Reslizumab was associated with a reduction in blood eosinophil counts compared with Placebo |
|  | 244 | Placebo | 0.624 (0.59) | 0.497 |  |  |
| Castro 2015 study 2^29^ | 232 | Reslizumab 3.0 mg/kg | 0.61 (0.412) | 0.045 | <0.0001 |  |
|  | 232 | Placebo | 0.688 (0.682) | 0.612 |  |  |
| Corren 2016^30^ | 398 | Reslizumab 3.0 mg/kg | 0.281(0-1.584) | NA | <0.0001 | Reslizumab significantly decreased blood eosinophils |
|  | 98 | Placebo | 0.277(0-1.288) | NA |  |  |
| Bjermer L 2016^31^ | 104 | Reslizumab 0.3 mg/kg | 0.648(0.1-3.7) | 0.29(0.28) | NA | Overall reductions in blood eosinophil levels were greater with reslizumab versus Placebo, with the greatest decreases observed with 3.0 mg/kg |
|  | 106 | Reslizumab 3.0 mg/kg | 0.592(0.1-2.3) | 0.063(0.28) | NA |  |
|  | 105 | Placebo | 0.601(0.1-3.7) | 0.566(0.28) |  |  |
| Laviolette 2013 cohort 1^32^ | 8 | Benralizumab 1 mg/kg | 0.15(0.1-0.6) | 0 (0-0) | <0.0001 | Benralizumab administration resulted in reduction of peripheral blood eosinophils |
|  | 5 | Placebo | 0.40 (0.2-0.7) | 0.40(0.3-0.5) |  |  |
| Laviolette 2013 cohort 2^32^ | 4 | Benralizumab 100 mg | 0.40 (0.2-0.9) | 0 (0-0) | NA |  |
|  | 5 | Benralizumab 200 mg |  |  |  |  |
|  | 5 | Placebo | 0.30 (0.1-0.6) | 0.20(0.1-0.8) |  |  |
| Castro 2014 group 1^33^ | 81 | Benralizumab 2.0 mg | 0.53 (0.33) | NA | NA | Benralizumab significantly decreased blood eosinophil counts compared with Placebo |
|  | 81 | Benralizumab 20 mg | 0.54 (0.28) | NA | NA |  |
|  | 82 | Benralizumab 100 mg | 0.56 (0.36) | NA | NA |  |
|  | 81 | Placebo | 0.53 (0.30) | NA |  |  |
| Castro 2014 group 2^33^ | 142 | Benralizumab 100 mg | 0.19 (0.12) | NA | NA |  |
|  | 143 | Placebo | 0.16 (0.09) | NA |  |  |
| Nowak 2015^34^ | 36 | Reslizumab 0.3 mg/kg | 0.259(0.511) | NA | NA | Administration of benralizumab produced a significant reduction in eosinophil counts at both dose |
|  | 36 | Reslizumab 1.0 mg/kg | 0.168(0.220) | NA | NA |  |
|  | 38 | plaocebo | 0.35(0.525) | NA | NA |  |
| Park HS 2016^35^ | 26 | Benralizumab 2 mg | 0.7427(0.9721) | NA | NA | The blood eosinophil count was depleted at all doses during the treatment period |
|  | 25 | Benralizumab 20 mg | 0.5832(0.4126) | NA | NA |  |
|  | 26 | Benralizumab 100 mg | 0.8154(1.0756) | NA | NA |  |
|  | 26 | Placebo | 0.5865(0.4434) |  |  |  |
| **Sputum eosinophils(%)** |  |  |  |  |  |  |
| Leckie 2000^18^ | 8 | Mepolizumab 2.5 mg/kg | 13.2(11.1) | 6.8 | 0.4454 | Mepolizumab at doses of 10 mg/kg caused significant changes in sputum eosinophils |
|  | 8 | Mepolizumab 10 mg/kg | 13.1(10) | 0.7 | 0.005 |  |
|  | 8 | Placebo | 11.1(11.5) | 12.8 |  |  |
| Haldar 2009^22^ | 29 | Mepolizumab 750 mg | 6.8(0.6)^*^ | 0.952^*^ | 0.002 | Mepolizumab therapy was associated with significant reductions in sputum eosinophils |
|  | 32 | Placebo | 5.46(0.75)^*^ | 2.78^*^ |  |  |
| Nair 2009^23^ | 9 | Mepolizumab 750 mg | 16.6(1.6-54.3) | 0.3(0-4.6) | 0.005 | A single infusion of mepolizumab was associated with a reduction in sputum eosinophils |
|  | 11 | Placebo | 4(0–35.3) | 5(1-9) |  |  |
| Pavord 2012^24^ | 153 | Mepolizumab 75 mg | 13.9(1.47)^#^ | NA | 0.6429 | In the subgroup of 94 patients who had sputum induction, the mepolizumab also caused decreases in sputum eosinophil counts compared with Placebo |
|  | 152 | Mepolizumab 250 mg | 8.1(1.79)^#^ | NA | 0.1577 |  |
|  | 156 | Mepolizumab 750 mg | 5.8(2.15)^#^ | NA | 0.0082 |  |
|  | 155 | Placebo | 6.8(2.01)^#^ |  |  |  |
| Castro 2011^28^ | 53 | Reslizumab 3.0 mg/kg | 10.7(1.7-67.6) | -84.7(–100 -315.9) | 0.0068 | Reslizumab showed significantly greater reductions from baseline in eosinophils in the induced sputum |
|  | 53 | Placebo | 8.5 (3.0-77.0) | -30.2(–96 -1480) |  |  |
| Laviolette 2013 cohort 1^32^ | 8 | Benralizumab 1 mg/kg | 5.7(4.3–11.0) | 4.5(0–16.5) | 0.04 | Benralizumab administration resulted in reduction of sputum eosinophils |
|  | 5 | Placebo | 13.0(4.8–31.0) | 20.8(2.5–33.3) |  |  |
| Laviolette 2013 cohort 2^32^ | 4 | Benralizumab 100 mg | 4.6(2.5–20.8) | 0.6(0–3.5) | NA |  |
|  | 5 | Benralizumab 200 mg |  |  |  |  |
|  | 5 | Placebo | 16.82.9–73.9) | 6.4(1.9–20.0) |  |  |

All data are median (minimum-maximum values) or mean (SD). *Values are geometric means (log SD)

^#^ Values are geometric mean on loge scale. NA: not applicable.
