# Supplementary material for: Efficacy and Safety of Anti-Interleukin-5 Therapy in Patients with Asthma: A Systematic Review and Meta-Analysis
Source: PLoS One. 2016 Nov 22;11(11):e0166833. doi: 10.1371/journal.pone.0166833 (PMC5119789; doi:10.1371/journal.pone.0166833)
Supplement: S3 Table — (DOCX) [file pone.0166833.s003.docx]

**S3 Table. The data of all outcomes in all RCTs**

|  |  | experiment | |  | control |  |
| --- | --- | --- | --- | --- | --- | --- |
|  | n | mean | SD | n | mean | SD |
| FEV_1_ L/sec |  |  |  |  |  |  |
| Flood-Page PT 2003 | 11 | 0.12 | 1.69 | 13 | -0.05 | 2.04 |
| Flood-Page P 2007 | 222 | 0.085 | 1.32 | 119 | 0.11 | 1.18 |
| Haldar 2009 | 27 | 0.06 | 0.38 | 29 | 0.12 | 0.38 |
| Nair 2009 | 9 | 0.3 | 0.9 | 10 | 0.1 | 0.78 |
| Pavord 2012 | 461 | 0.12 | 0.46 | 155 | 0.06 | 0.47 |
| Ortega 2014 | 385 | 0.17 | 0.46 | 191 | 0.03 | 0.47 |
| Castro 2011 | 52 | 0.18 | 0.372 | 52 | -0.08 | 0.413 |
| Castro 2015 study 1 | 245 | 0.235 | 0.73 | 244 | 0.109 | 0.8 |
| Castro 2015 study 2 | 232 | 0.201 | 0.78 | 232 | 0.111 | 0.67 |
| Corren 2016 | 394 | 0.225 | 0.205 | 97 | 0.187 | 0.393 |
| Bjermer L 2016 | 203 | 0.26 | 0.56 | 103 | 0.126 | 0.56 |
| Castro 2014 group 1 | 244 | 0.18 | 0.46 | 80 | 0.04 | 0.46 |
| Castro 2014 group 2 | 140 | 0.06 | 0.33 | 142 | -0.01 | 0.3 |
| Nowak 2015 | 67 | 0.24 | 0.75 | 36 | 0.35 | 0.67 |
| Bleecker E R 2016 | 797 | 0.33 | 0.57 | 407 | 0.21 | 0.584 |
| FitzGerald J M 2016 | 886 | 0.28 | 0.62 | 440 | 0.2 | 0.645 |
| FEV1% of predicted value |  |  |  |  |  |  |
| Nair 2009 | 9 | 3.7 | 16.36 | 10 | 3.8 | 18.54 |
| Bel 2014 | 69 | 3.68 | 7.45 | 66 | 0.03 | 8.15 |
| Ortega 2014 | 385 | 6.27 | 19.6 | 191 | 3.18 | 13.8 |
| Kips JC 2003 | 18 | 9.47 | 9.98 | 8 | 4 | 13.28 |
| Castro 2011 | 52 | 6.19 | 11.757 | 52 | -2.44 | 12.927 |
| Nowak 2015 | 67 | 8.1 | 20.2 | 36 | 10.5 | 17 |
| Park HS 2016 | 77 | 14.16 | 26.8 | 26 | 12 | 22.1 |
| Morning PEFR L/min |  |  |  |  |  |  |
| Flood-Page PT 2003 | 11 | 3 | 67.2 | 13 | -11.5 | 79 |
| Flood-Page P 2007 | 222 | 17.6 | 179.3 | 119 | 9.17 | 184.1 |
| Nowak 2015 | 67 | 39.3 | 108.3 | 36 | 57.1 | 102.2 |
| Park HS 2016 | 77 | 15.5 | 26.8 | 26 | 9.4 | 16.5 |
| Histamine PC20 mg/ml |  |  |  |  |  |  |
| Leckie 2000 | 16 | -0.85 | 1.76 | 8 | 0 | 1.63 |
| Flood-Page PT 2003 | 11 | 0.45 | 19.7 | 13 | 0.64 | 2.23 |
| Haldar 2009 | 29 | -0.06 | 5.5 | 32 | -0.66 | 7.58 |
| AQLQ |  |  |  |  |  |  |
| Haldar 2009 | 29 | 0.55 | 0.81 | 32 | 0.19 | 0.51 |
| Pavord 2012 | 461 | 0.83 | 1.11 | 155 | 0.71 | 1.12 |
| Castro 2015 study 1 | 245 | 1.09 | 1.187 | 244 | 0.79 | 1.182 |
| Castro 2015 study 2 | 232 | 1.12 | 1.22 | 232 | 0.89 | 1.24 |
| Bjermer L 2016 | 195 | 1.1 | 1.83 | 101 | 0.779 | 1.83 |
| Castro 2014 | 182 | 1.17 | 1.28 | 88 | 0.96 | 1.33 |
| Nowak 2015 | 67 | 1.79 | 1.24 | 35 | 1.77 | 1.48 |
| Bleecker E R 2016 | 542 | 1.5 | 0.98 | 267 | 1.26 | 0.99 |
| FitzGerald J M 2016 | 480 | 1.51 | 1.02 | 248 | 1.31 | 1.04 |

| asthma exacerbation rate RR | LCI | UCI | logRR | SElog |
| --- | --- | --- | --- | --- |

| Flood-Page P 2007 | 0.85 | 0.51 | 1.43 | -0.16 | 0.27 |
| --- | --- | --- | --- | --- | --- |
| Haldar 2009 | 0.82 | 0.61 | 1.09 | -0.20 | 0.15 |
| Nair 2009 | 0.24 | 0.07 | 0.84 | -1.43 | 0.64 |
| Pavord 2012 | 0.54 | 0.47 | 0.61 | -0.62 | 0.06 |
| Bel 2014 | 0.69 | 0.51 | 0.94 | -0.37 | 0.16 |
| Ortega 2014 | 0.53 | 0.43 | 0.65 | -0.63 | 0.10 |
| Kips JC 2003 | 1.33 | 0.16 | 10.94 | 0.29 | 1.08 |
| Castro 2011 | 0.42 | 0.14 | 1.27 | -0.87 | 0.56 |
| Castro 2015 study 1 | 0.69 | 0.57 | 0.85 | -0.37 | 0.11 |
| Castro 2015 study 2 | 0.56 | 0.43 | 0.73 | -0.58 | 0.14 |
| Castro 2014 group 1 | 0.79 | 0.63 | 1 | -0.24 | 0.12 |
| Castro 2014 group 2 | 0.76 | 0.6 | 0.97 | -0.27 | 0.12 |
| Nowak 2015 | 0.91 | 0.59 | 1.41 | -0.09 | 0.22 |
| Bleecker E R 2016 | 0.62 | 0.49 | 0.77 | -0.48 | 0.11 |
| FitzGerald J M 2016 | 0.66 | 0.56 | 0.77 | -0.42 | 0.08 |

| adverse events | events | total | events | total |
| --- | --- | --- | --- | --- |
| Nair 2009 | 2 | 9 | 2 | 11 |
| Pavord 2012 | 161 | 461 | 62 | 155 |
| Bel 2014 | 57 | 69 | 61 | 66 |
| Ortega 2014 | 313 | 385 | 158 | 191 |
| Castro 2011 | 38 | 53 | 42 | 53 |
| Castro 2015 study 1 | 197 | 245 | 206 | 243 |
| Castro 2015 study 2 | 177 | 232 | 201 | 232 |
| Corren 2016 | 218 | 395 | 74 | 97 |
| Bjermer L 2016 | 120 | 206 | 66 | 105 |
| Laviolette 2013 cohort 1: | 5 | 8 | 5 | 5 |
| Laviolette 2013 cohort 2: | 6 | 9 | 5 | 5 |
| Castro 2014 | 277 | 385 | 143 | 221 |
| Park HS 2016 | 73 | 77 | 25 | 26 |
| Bleecker E R 2016 | 574 | 797 | 311 | 407 |
| FitzGerald J M 2016 | 642 | 866 | 342 | 440 |
